# A Machine Learning Approach for Using the Postmortem Skin Microbiome to Estimate the Postmortem Interval

**DOI:** 10.1371/journal.pone.0167370

**Published:** 2016-12-22

**Authors:** Hunter R. Johnson, Donovan D. Trinidad, Stephania Guzman, Zenab Khan, James V. Parziale, Jennifer M. DeBruyn, Nathan H. Lents

**Affiliations:** 1 Department of Mathematics and Computer Science, John Jay College, The City University of New York, New York, NY, United States of America 10019; 2 Department of Sciences, John Jay College, The City University of New York, New York, NY, United States of America 10019; 3 Department of Biosystems Engineering & Soil Science, University of Tennessee, Knoxville, TN, United States of America, 37996; Laboratory of Bacterial Pathogenesis and Immunology, UNITED STATES

## Abstract

Research on the human microbiome, the microbiota that live in, on, and around the human person, has revolutionized our understanding of the complex interactions between microbial life and human health and disease. The microbiome may also provide a valuable tool in forensic death investigations by helping to reveal the postmortem interval (PMI) of a decedent that is discovered after an unknown amount of time since death. Current methods of estimating PMI for cadavers discovered in uncontrolled, unstudied environments have substantial limitations, some of which may be overcome through the use of microbial indicators. In this project, we sampled the microbiomes of decomposing human cadavers, focusing on the skin microbiota found in the nasal and ear canals. We then developed several models of statistical regression to establish an algorithm for predicting the PMI of microbial samples. We found that the complete data set, rather than a curated list of indicator species, was preferred for training the regressor. We further found that genus and family, rather than species, are the most informative taxonomic levels. Finally, we developed a *k*-nearest- neighbor regressor, tuned with the entire data set from all nasal and ear samples, that predicts the PMI of unknown samples with an average error of ±55 accumulated degree days (ADD). This study outlines a machine learning approach for the use of necrobiome data in the prediction of the PMI and thereby provides a successful proof-of- concept that skin microbiota is a promising tool in forensic death investigations.

## 1 Introduction

The human body is inhabited by a vast number of microorganisms, which have occupied every conceivable ecological niche. Recent advances in sequencing has resulted in a great deal of research focused on the human microbiome. [1] In particular, the microbiota of the skin is increasingly the subject of research into inter-personal differences and microbe-host interactions, revealing that microbial communities differ between individuals and between different sites on the body. [2] Compared to that of the gut and oral cavity, the skin microbiome appears to be more influenced by the host environment. [3] It is also becoming apparent that skin microbial communities play a role in many diseases, including obesity, diabetes, cancer and chronic inflammatory disease. [4] While the skin microbiome of living individuals has recieved attention, we know much less about the fate of these microbial communities after host death. These microbes are likely strongly influenced by the decomposition environment, which includes insect colonization and changes in soft tissue chemistry.

Several recent reports have aimed to describe the postmortem human microbiome. Not surprisingly, the various microbial communities that colonize the human person change considerably following the death of the host as the chemical and biological milieu changes in almost every conceivable way. [5] The primary goal of research into the postmortem microbiome, or necrobiome, is to aid in death investigations by providing a means to reliably estimate the postmortem interval (PMI). Current methods of estimating the PMI of a deceased human person discovered in an uncontrolled environment are quite crude, involving subjective physical inspection of the cadaver for early-phase decomposition and insect colonization in later-phase decomposition. However, these techniques are notoriously unreliable. The use of entomology, in particular, is confounded by temperature, weather conditions, seasonal variation, geographic location, and many other factors, both known and unknown. The high variability of these PMI estimation techniques makes it clear that additional approaches are needed. Microbiome-based estimates may prove particularly useful in cases where insects are absent or delayed, such as indoors, burials, or in colder temperatures.

In a 2013 study Metcalf, et al. endeavored to discover a so-called “microbial clock” to provide estimates of the postmortem interval in mice. [6] Impressively, the model that they developed was accurate to within three days of error over a period of 48 days of decomposition. It is worth noting, however, that the ambient conditions were held steady during the course of the experiment and insects were excluded in order to reduces sources of environmental variability. The controlled environment is an important first step, but favors construction of a robust predictive model at the expense of attempting to replicate the conditions when the model might actually be used, that is, human decedents discovered after an unknown period of time in uncontrolled environments.

A study by Pechal, et al. reported on the usefulness of monitoring the succession of bacterial taxa during the course of decomposition of pigs in an uncontrolled environment. [7] In that study, they were able to build a statistical model using metagenomic sampling that explained nearly 95% of the microbiome changes that occurred through the course of decomposition. This convincingly demonstrated that a data analytics approach can overcome the inevitable noised introduced by sampling from an outdoor environment. An earlier study by the same group described temperate-zone seasonal variations of the swine necrobiome, an important consideration for forensic investigations throughout the global North. [8] Seasonal variations in the decomposition microbiome of pigs were also reported by Carter, et al., who also reported an important contribution of soil-derived microbes to the decomposition ecosystem of the pig cadavers. [9]

Other interesting work on the necrobiome has probed how microbial communities in cadaver tissue and the soil merge into one dynamic system. Studies by Finley, et al., and Cobaugh, et al. have reported that cadaver-derived microbes can be detected in the nearby soil for up to a year and possibly much longer. [10, 11] Importantly, the detection of microbes in nearby soil appears to follow a steady progression and could prove fruitful for forensic estimation of the postmortem interval, even long after a body has been removed the scene.

Recently, Hauther, et al., repeatedly sampled bacteria from the large intestine of 12 cadavers as they decomposed in outdoor environmental conditions. [12] This study performed quantitative analysis of bacterial communities using the 16S rRNA gene for phylogenetic identifications and identified three specific genera of bacteria that show specific promise as quantitative indicators of the postmortem interval.

In the largest and most comprehensive study on the human necrobiome to date, Metcalf, et al. used machine learning methods to characterize how the microbes derived from both the soil and the decomposing human or mouse cadaver assemble into a decomposition ecosystem with a predictable succession of bacterial and fungal organisms. [8] Using mouse cadavers in laboratory conditions and human cadavers in uncontrolled outdoor conditions, this study found that patterns of microbial succession were surprisingly independent of soil type and seasonal effects. In addition, researchers found many microbial taxa that are active in a similar schedule in both the human and mouse conditions. This implies that the sudden influx of carrion-derived nutrients into the soil initiates a common biological chain of events at the microbial level, regardless of the mammal species from which the carrion derives. This commonality bodes well for the forensic utility of the necrobiome.

To date, most reports on the microbiome have focused on the communities of the GI tract, as it is the site of the richest diversity of microflora to begin with. However, we propose that that the skin microbiome offers several advantages as a site-of-interest for research into the postmortem microbiome. Specifically, we chose the aural and nasal cavities for our study, which are included as sites of interest in the NIH Human Microbiome Project. [13] These two niches are each unique environments in the body but are also accessible and non-invasive to sample. Using the ear and nasal cavities would offer distinct advantages during an active criminal investigation of a crime scene, as it would leave the cadaver essentially undisturbed.

In this study, we sampled the bacterial communities in the ear and nasal canals of 17 cadavers, four of them repeatedly, throughout the course of surface decomposition and analyzed those communities with 16S rRNA gene amplicon sequencing. Statistical analysis at all taxonomic levels was used in a machine learning approach toward development of a computational model for prediction of the postmortem interval. To that end, we were successful in constructing a *k* = 4 nearest-neighbor regression model which accurately predicted the true postmortem interval to within 55 accumulated degree days (ADD), or two days at an average temperature of 27.5°C. We were also able to identify the bacterial taxa that are most informative of our predictive model of decomposition.

## 2 Materials and Laboratory Methods

### 2.1 Sample collection and purification of DNA

All samples were collected from cadavers placed at the Anthropological Research Facility (ARF) at the University of Tennessee at Knoxville. The use of deceased human subjects at the ARF does not require IRB approval as the bodies were donated for research purposes to the facility. In addition, this research was reviewed and approved by the Internal Review Board of the City University of New York as part of larger project (protocol #514576). The ARF is a preserved temperate deciduous forest with well-drained fine textured clayey soils. All cadavers were placed on the surface of the soil and allowed to decompose naturally. Bodies were placed in a prostrate position, unclothed, and loosely covered to reduce mass scavenging by large animals. A total of 144 sample swabs were taken from a total of 21 cadavers, most as a single collection event for each cadaver. However, four of these cadavers were swabbed repeatedly through the course of decomposition, starting at placement and continuing every 2–3 days until the tissues were too decomposed to access the ear and/or nose. Data from only these four cadavers was used in the early phase of our computational analysis when the models that were best suited for our data were selected, as explained below. Subsequent computational modeling included data from all suitable samples.

During sample collection, aseptic technique was utilized as much as possible. Because the bodies were placed prostrate, the heads are turned, facing one side of the other. For each cadaver, the nostril and ear canal chosen for sampling was that on the side of the face facing away from the ground, preventing or minimizing the sampling of bacteria from the soil and nearby cadavers. Just prior to each sample collection, the extreme tip of a fresh, sterile, Cap-Shure™ swab was briefly dipped into sterile phosphate-buffered saline (obtained 1X from Fisher Scientific). Then, the swab was placed into the nostril or ear canal until the entire cotton swab was inside the canal. The swab was gently pressed against the outside wall before moving the swab in a circular motion for two complete rotations. The swab was then placed back into its collection tube, which was itself placed back into its plastic wrapping, separately, and then into a sterile sample collection bag. Sample bags were kept at -20°C until DNA extraction was performed.

### 2.2 DNA Extraction and Quantitation

DNA was extracted using the PowerLyzer PowerSoil DNA Isolation Kit (MoBio Laboratory) precisely according to the manufacturer’s protocol. Final purified DNA was eluted in a final volume of 100 μL. DNA concentration was determined by measuring the absorbance of each sample at 260 nm using the NanoDrop 2000 Spectrophotometer (Thermo Scientific). Only samples harboring at least 1 ng/μL DNA and yielded a clean spectrogram with a peak at 260 nm were included in subsequent amplification steps.

### 2.3 DNA Amplification, Quantification and Sequencing

Preparation of sample amplification for sequencing was completed by following the 16S Metagenomic Sequencing Library Preparation protocol by Illumina. Briefly, the V3 and V4 regions of the 16S rRNA gene were amplified using universal degenerate primers in the provided kit. Procedures were exactly according to protocol with the following exceptions: volumes used were 7.5 μL template DNA, 2.5 μL Amplicon PCR Forward Primer (1 M), 2.5 μL Amplicon PCR Reverse Primer (1*μ*M), and 12.5*μ*L 2x KAPA HiFi HotStart ReadyMix for a total of 25*μ*L. This modification was to increase the DNA concentration used per sample, since some samples were too low to follow the recommended protocol precisely.

Following the amplification step, 2 μL of each sample was run in a 1.5% agarose gel to ensure that the PCR was successful. Only those samples that yielded a clean PCR product at 550bp were included in future steps. PCR clean up was performed using the Agencourt AMPure XP beads kit by Beckman Coulter, following the provided protocol precisely. Final purified DNA was eluted in a volume of 50 μL and [DNA] was determined. Samples with less than 15 ng/μL were concentrated down to ≈ 25 μL using a speedvac before proceeding to the index PCR.

Next, 25 μL of the purified amplicon PCR product was barcoded by index PCR using the Nextera XT Index Kit from Illumina, as per provided protocol. Following PCR cleanup (again using the AMPure XL kit), 2 μL of each barcoded PCR product was subjected to agarose gel electrophoresis to verify integrity of each sample through visualization of a 630bp band.

The barcoded DNA amplicons were pooled together and delivered to the Genome Technology Center at NYU Langone Medical Center for next-generation 16S metagenomic sequencing using the MiSeq platform (Illumina). Compiled sequence libraries were analyzed and phylogentically classified using the BaseSpace program (Illumina). Spreadsheets with absolute numbers of sequence reads for each taxon in each sample were extracted and transformed to relative abundance measures as explained below.

### 2.4 Calculation of Accumulated Degree Days

Because the events of tissue decomposition are equally dependent on time and temperature, we calculated the accumulated degree days (ADD) for each sample, with each 24 hour period postmortem counting as an equivalent to its average temperature in Celsius, as explained in Michaud, et al. [14] The following assumptions were made in these calculations. First, because cadaver placements at the ARF facility take place between 11:00A.M. and 1:00P.M., and all swabs are taken around that same time, we converted each day into two-half days, halving the average temperature for each day. Secondly, all cadavers were kept at 3°C for all days between the date of death and placement at the facility. Thirdly, if a cadaver was frozen, those days counted as zero toward the cumulative ADD value. Fourthly, average temperature listed for Knoxville, Tennessee, as recorded in Weather Underground (www.wunderground.com) was used as a proxy measurement for the average temperature at the ARF facility, which does not track and archive precise local temperature.

## 3 Computational Methods

### 3.1 Data Transformations

The data for this project was produced by collecting a series of bacterial swabs from the ear and nasal canals of cadavers undergoing active decomposition in uncontrolled environmental conditions. Each swab is annotated with a time-since-death measurement that accounts for both temperature and time elapsed in a single variable, accumulated degree days (ADD). The DNA collected in each swab was subjected to quantitative sequencing of the 16S rDNA gene, which allowed detection of individual species and subsequent calculation of its relative abundance in the entire sample. Thus, the raw data consists of percent-abundance of each sequence (representing a bacterial species) and the ADD to which the cadavers were exposed at the time of the swab. In order to study these data as a regression problem, we set the independent variables as the relative abundance of the species (or higher taxa) in each collection swab and the dependent variable as the ADD value for that swab. The aim of our regression analysis was to observe if the dependent variable, ADD, can be determined as a function of the independent variables.

In addition to species classification, each sequence read was also assigned to its proper kingdom, phylum, class, order, family, and genus. Mathematically, the species measurements may be considered to be the raw data, with data corresponding to higher taxonomic levels understood as transformations of the original data. In the parlance of machine learning, this is a form of feature extraction. Considering the data at higher taxa clusters the species into similar kinds, preserving some amount of information, while reducing the number of independent variables, and possibly increasing the effectiveness of the regression. [15]

We regard the data as matrices *X* in which rows correspond to instances–swabs–and columns correspond to features—organisms (or taxa). The symbol *X* can be regarded as a multidimensional independent variable, for which there is a corresponding dependent vector variable *y*. If *X* is *m* × *n* then *y* is a column vector of length *m*, where row *i* of *X* determines (at least partly) the ADD value found in row *i* of *y*. There are a number of different possible matrices *X*, depending on which swabs, which organisms, and which taxonomic levels are considered. For instance, we may consider only nose swabs and restrict our attention to a special subset of organisms at the phylum level, and attempt a regression of *y* from the resulting matrix *X*. In another case, *X* may include both ear and nose swabs, and refer to the data at the class level. We thus consider several different transformations of our data to see which transformation performs best with respect to the problem of determining *y* from *X*. The main differences analyzed refer to whether *X* contains only ear data, only nose data, or both jointly, and whether all organisms are considered, or only a special curated subset, as described below.

### 3.2 Selection of Curated Data

**Fig 1:**
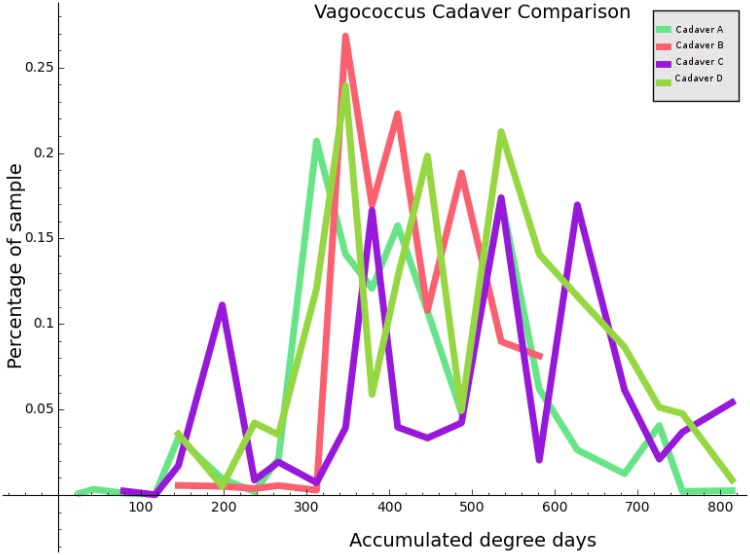
A plot of ADD versus percentage composition for the bacterial genus *Vagococcus*. Each of the four sample cadavers has a corresponding curve, as indicated in the legend.

For data that came from the four cadavers that were sampled repeatedly throughout decomposition, we also attempted to refine our data matrices into two different types: curated and non-curated. The non-curated data matrices *X* have one column corresponding to each organism occurring a nonzero number of times in some ear sample, or some nose sample (the same organism is frequently represented twice–once for the ear and once for the nose). The curated data employs a reduced set of organisms (both for ear and for the nose). The pruning of organisms was done manually through visual inspection, considering factors such as the correlation coefficient of the organism with the dependent variable, and the coherence of any correlation across multiple cadavers. To facilitate this process, code was written to plot ADD against percentage composition for each taxon, producing 5243 individual plots. In these images, data was color coded by cadaver, allowing an intuitive judgement to be made regarding the agreement across cadavers, as well as the plausibility of a functional relationship between ADD and percent composition. Fig 1 provides an example of an image of this type.

A separate pruning was done at each level of the taxonomic hierarchy. The effect is that the curated data set has many fewer columns than the non-curated data, though they have an equal number of rows. The curated data is also higher quality in the sense that the selected taxa have unusually high agreement across cadavers as well as higher magnitude correlation values with respect to ADD and percent composition. Table 1 summarizes the dimensions of our data matrices.

**Table 1:** Summary of data matrix dimensions for joint data (swabs for both ear and nose). The number of rows in each table is 67 for all data, and the number of columns is the number of organisms, as shown. We also provide the logarithm of the number of columns in each dataset, for later reference.

Dataset	Number of organisms	Logarithm
KINGDOM_NONCURATED_JOINT	6	1.792
PHYLUM_CURATED_JOINT	7	1.946
CLASS_CURATED_JOINT	13	2.565
ORDER_CURATED_JOINT	22	3.091
FAMILY_CURATED_JOINT	30	3.401
PHYLUM_NONCURATED_JOINT	52	3.951
GENUS_CURATED_JOINT	54	3.989
SPECIES_CURATED_JOINT	65	4.174
CLASS_NONCURATED_JOINT	106	4.663
ORDER_NONCURATED_JOINT	213	5.361
FAMILY_NONCURATED_JOINT	478	6.170
GENUS_NONCURATED_JOINT	1264	7.142
SPECIES_NONCURATED_JOINT	3130	8.049

The exact set of organisms used for each level for both non-curated and curated cases can be found in our supplementary materials (http://bit.ly/2f4ltDH). When the data matrix *X* is built from a spreadsheet, it contains integers for entries, with entry *x*_*i*,*j*_ reflecting the absolute number of instances for organism *j* detected in sample *i*. Because this number may depend on, for example, the richness of the swab offered to the analyzer, it is normalized in the following way. For each row *i* of *X*, we compute *s*_*i*_ = *Σ*_*j* ≤ *n*_ *x*_*i*,*j*_, and replace $\bar{x_{i}}=\langle x_{i,1},x_{i,2},\ldots ,x_{i,n}\rangle$ with $\langle \frac{x_{i,1}}{s_{i}},\frac{x_{i,2}}{s_{i}},\ldots ,\frac{x_{i,n}}{s_{i}}\rangle$. Note that this form of normalization depends only on the row (*i.e.* sample), and can be done to new data without information from *X*. We do not perform column-based normalization (so-called *feature normalization*) manually, but this may be done in some of the algorithms we use for regression. Consult our code in the supplementary materials for details.

### 3.3 Calculation of Microbial Diversity

In our discussion of diversity, we will use the following quantity to measure diversity of the microbial communities in each swab: [16]

$${}^{q}D=\frac{1}{\sqrt[{q-1}]{\sum_{i=1}^{S}p_{i}p_{i}^{q-1}}}.$$

Here the variable *S* refers to species richness (the number of species present) and *p*_*i*_ is the proportion of the population accounted for by the *i*^*th*^ species. This quantity, which depends on a nonnegative free variable *q*, can be understood as the reciprocal of the weighted power mean (with power *q* − 1) of the proportions *p*_*i*_ of the various species, where the weight of species *i* is *p*_*i*_.

Intuitively, when *q* is small, ^*q*^*D* provides a formalization of diversity which emphasizes species richness, whereas when *q* is large, equal representation of species in the environment (*i.e.* equitability) becomes more influential. In fact when *q* = 0, ^*q*^*D* is simply the species richnes. Note also that special values of *q* cause ^*q*^*D* to specialize to a number of well known diversity indices. For example, if *q* = 2, then ^*q*^*D* is Simpson’s diversity index $D=\frac{1}{\sum_{i=1}^{S}p_{i}^{2}}$, and while ^*q*^*D* is undefined for *q* = 1, it is the case that lim_*q* → 1_^*q*^ *D* = exp(*H*′) where *H*′ denotes the Shannon index defined by $H^{\prime }=-\sum_{i=1}^{S}p_{i}\ln\left(p_{i}\right)$.

It is not necessary that ^*q*^*D* be computed literally with respect to species. For example the *p*_*i*_ can be sample composition percentages at the level of phylum, genus, *etc*, in which case ^*q*^*D* represents diversity at the level of the appropriate taxon. For a fixed *q*, higher values of ^*q*^*D* correspond to higher levels of diversity.

### 3.4 Regression Analysis

To analyze the data, several regression techniques were considered. To evaluate the effectiveness of the regressors, we used a machine learning approach, splitting the data randomly into two mutually exclusive groups called a training set and a testing set. The train/test split depends only on *m* and is the same for every taxon. The training set is selected uniformly at random to comprise 80% of the instances. The testing set is then taken as the remaining 20% of instances. [17] The regressors we employed in our analysis were the following (as implemented by the scikit-learn project, version 0.17): [18] Support Vector Regression (SVR) [19], K-neighbors Regression (KNR) [15], Ridge Regression (RR) [20], Lasso Regression (LR) [15], Elastic Net Regression (ENR), Random Forest Regression (RFR) [21], and Bayesian Ridge Regression (BRR) [20].

Describing each of these regressors in detail is beyond the scope of this report, but a few general remarks may be helpful. The regressors SVR, RR, RFR, LR and BRR all fit a collection of linear coefficients to the data. They differ from one another and from simple linear regression in the objective function and their constraints, which are optimized during the fitting process. All of these regressors include some kind of *regularization* which is a penalty assigned in the objective function to complicated solutions. [15] Usually this amounts to penalizing coefficients which, when viewed as a vector, have a large norm with respect to either the L1 or L2 norms (the Manhattan metric and Euclidean metric, respectively). The values that control the degree to which complicated solutions are penalized are known as the *hyperparameters* for the regressor. [15] Fitting the hyperparameters to the data is important to avoid overfitting. Note that for the non-curated dataset, we have *n* > *m* in all cases except for kingdom and phylum, necessitating some form of regularization to avoid trivial solutions.

The KNR regressor is a simple instance-based approach, which means that no parameters are fit to the data. [15] Rather, the data itself is stored directly and used to predict the values for new instances. In the KNR case, this happens simply by averaging the known ADD for the *k* most similar training points for some new (test) instance. The measure of similarity may be Euclidean distance (when the data are regarded as vectors) or something more exotic. Though the KNR regressor does not have parameters, it does have hyperparameters, namely *k* and specifics of the algorithm used, which might specify the meaning of “similarity” or weight the training vectors in certain ways in an attempt to improve performance. An interesting corollary of the instance-based nature of KNR is that the training accuracy (the accuracy of the predictions the model makes on the training set) is always perfect.

The RFR regressor is an ensemble method, meaning it employs a voting approach based on a family of simpler regressors. In the case of RFR, the simpler family of regressors consists of decision trees. This model neither has parameters nor is it instance-based. A more lengthy discussion can be found in the references. [21]

## 4 Results

### 4.1 Effect of Decomposition on Microbial Diversity

**Fig 2:**
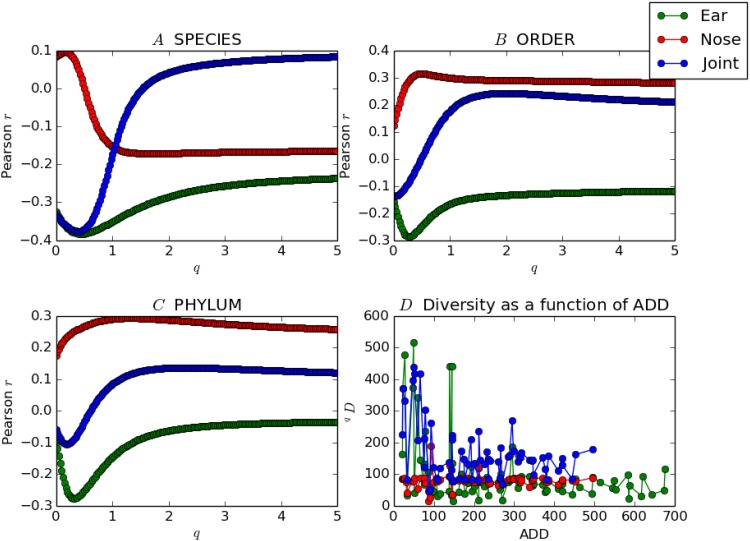
The images *A*, *B*, and *C* show how the correlation between ^*q*^*D*(*X*) and *y* depends on the choice of *q* and the dataset *X*. The image *D* shows how diversity changes with ADD for the ear, nose and joint datasets (*q* = 0.4).

We began the analysis of our data by inquiring if there was a useful relationship between ADD and sample diversity. For a data matrix *X*, recall that the rows of *X* represent population samples, and any given row naturally corresponds to *p*_1_,*p*_2_, …, *p*_*S*_ as in the definition of ^*q*^*D*. Then if ^*q*^*D*(*X*) denotes the column vector whose rows are the diversity values with respect to ^*q*^*D* of the corresponding rows of *X*, we asked if there was any correlation between ^*q*^*D*(*X*) and *y*. We used Pearson’s correlation coefficient to measure the strength of such a relationship for a number of values of *q* and several different taxa. Consider Fig 2, below.

Panels *A*, *B*, and *C* in Fig 2 show Pearson’s *r* as a function of *q* for the species, phylum, and order datasets, respectively. Each contains three curves, corresponding to whether *X* contains data taken only from nose swabs, only from ear swabs, or a combination of both (joint). For most datasets, diversity decreased as ADD increases for the microbial communities found in the ear swabs, as is the case with data from both the ear and the nose swabs considered jointly. Using data from the nose swabs alone yields weaker correlations for the most part, and diversity tends to have a positive (increasing) relationship to ADD.

Panel *D* of Fig 2 shows diversity (*q* = 0.4) as a function of ADD for the species data. Note that for ear-only data, the plot is predominantly decreasing for the first 100 ADD, after which it becomes comparatively constant. The nose-only data, by contrast, yields a curve that is approximately constant for this choice of taxon and *q*. It should be noted that in computing the diversity of the joint data, if the same organism occurs both in the ear and in the nose, this is counted as two taxa rather than one.

The lowest value attained for the correlation coefficient is *r* = −0.425 for curated, joint ear and nose, family-level data, with an optimal choice of *q* = 0.226. The highest value is *r* = 0.35 for noncurated, nose-only, class-level data for an optimal choice of *q* = 0.50. The *p* values for these *r* are *p* = 0.00033 and *p* = 0.0033, respectively. Table 2 displays the *r* coefficients with the lowest *p* values for each dataset, as well as the associated *q* values.

**Table 2:** The most significant correlation found between ^*q*^*D*(*X*) and *y* for each dataset *X*, and the optimizing *q* value. Kingdom data is omitted.

	Noncurated		Curated	
*X*	*r*	*q*	*r*	*q*
PHYLUM JOINT	0.137	2.312	0.187	0.477
PHYLUM EAR	−0.277*	0.327	0.141	0.779
PHYLUM NOSE	0.294*	1.332	0.181	0.050
CLASS JOINT	0.240	1.608	0.239	0.427
CLASS EAR	−0.274*	0.226	0.243*	0.352
CLASS NOSE	0.349**	2.739	0.163	1.759
ORDER JOINT	0.242*	1.985	−0.331*	0.879
ORDER EAR	−0.287*	0.276	−0.227*	1.935
ORDER NOSE	0.314*	0.503	0.219	0.025
FAMILY JOINT	0.234	2.261	−0.425***	0.226
FAMILY EAR	−0.330**	0.377	−0.421***	0.427
FAMILY NOSE	0.345**	5.000	0.321*	0.427
GENUS JOINT	−0.264*	0.276	−0.373**	0.251
GENUS EAR	−0.371**	0.427	−0.355**	0.377
GENUS NOSE	0.345**	5.000	0.299*	0.251
SPECIES JOINT	−0.377**	0.402	0.296*	0.000
SPECIES EAR	−0.383***	0.452	−0.249*	0.578
SPECIES NOSE	−0.171	1.709	0.309*	0.126

*** *p* < 0.0005,

** *p* < 0.005,

* *p* < 0.05

### 4.2 Analysis of Various Regressors by Cross-Validation

In order to evaluate the relative effectiveness of the regressors chosen for analysis, each first underwent a process of hyperparameter optimization, which was based solely on the training portion of the dataset. The method used was grid search combined with 10× cross-validation. In the following discussion, a *model* is a regressor together with a choice of hyperparameters.

In the 10× cross-validation process, the training data *T* is partitioned into 10 equally sized subsets *t*_1_, *t*_2_, …, *t*_10_. For each *i* ∈ {1, 2, 3, …, 10}, the model under consideration *M* is trained on *T*\*t*_*i*_ and tested on *t*_*i*_, to yield a performance score *p*_*i*_ (in units of mean absolute error). Here “training” means the same thing as “fitting” and “testing” means the same thing as “predicting”. The values *p*_*i*_ were then averaged to yield a final measure of the performance of the model, which we refer to as the training error based on 10× cross-validation.

For each of the regressors SVR, LR, KNR, RR, ENR, RFR and BRR, 10× cross-validation on the training set in combination with grid-search was used to tune the hyperparameters. The term *grid search* refers to the technique of fixing, for each regressor *R* and each corresponding hyperparameter *h*_*R*_ of *R*, a finite range of potential values *V*_*h*_*R*__ for *h*_*R*_. The cartesian product of the *V*_*h*_*R*__, where *h* ranges over the hyperparameters of *R*, determines a grid-like space of models all based on the regressor *R*. The grid-search algorithm tries each model in this space and selects the model with the best cross-validation score (*i.e.* lowest error) on the training set. We regard the resulting model as an instance of *R* with “tuned” hyperparameters.

Tuned models were ranked according to their cross-validation score on the training set. We then retrained these models on all of the training data, and applied the same models to the testing set. The resulting error rate provides an independent validation of the model, and an unbiased estimate of true accuracy. To approximate the best error rate achievable, we selected the combination of regressor, hyperparameters, and data transformation that gave the lowest cross-validation error on the training set. The error of this model on the test set (after retraining) provides an estimate of the accuracy of our method. [15]

We also singled out the model with lowest testing error (without consideration of performance on the training set). The model with lowest error on the test set is offered as the model most likely to perform well on unseen data. In this case we used our entire data set for model selection, not error estimation. [17]

There were 69 nose samples in our dataset. The mean ADD found in the nose data is 210. These samples were taken at roughly uniform intervals from 0 to about 500 ADD. Table 3 outlines the results of our regression attempts using nose samples alone.

**Table 3:** The top ten models as ranked by cross-validation error on the training data when restricted to nose data are shown here. The error units in columns 1 and 4 are mean absolute error. The values in the NRMSE column are root mean squared error on the test set, divided by the mean ADD over all nose data.

Training error	NRMSE	Regressor	Test error	Dataset
74.03	0.84	KNR	138.45	SPECIES_NONCURATED_NOSE
76.60	0.67	SVR	121.15	ORDER_NONCURATED_NOSE
79.13	0.58	KNR	103.55	GENUS_NONCURATED_NOSE
79.53	0.69	KNR	116.66	ORDER_NONCURATED_NOSE
81.93	0.76	SVR	122.38	PHYLUM_NONCURATED_NOSE
82.16	0.57	SVR	102.58	SPECIES_NONCURATED_NOSE
84.57	0.61	KNR	114.67	CLASS_NONCURATED_NOSE
84.97	0.47	SVR	85.16	GENUS_NONCURATED_NOSE
86.76	0.53	KNR	87.95	SPECIES_CURATED_NOSE
86.94	0.71	KNR	128.13	FAMILY_NONCURATED_NOSE

The first column in Table 3 provides the cross-validation error of the model on the training set, in units of mean absolute error. As explained in the Methodology section, we used 10×cross-validation on the training set to rank our models and therefore Table 3 proceeds from the best models at the top, to the least useful models at the bottom. The column labeled NMRSE is computed by dividing the root mean squared error of the model on the test set, and dividing by the mean of *y*. This normalized value is intended to provide a measure of model quality that is comparable across data sets. The fourth column of Table 3 shows the error of the model on the testing set in units of mean absolute error.

In order to put the results shown in Table 3 in context, it is useful to compare the performance of the models shown to the performance of a so-called “dummy” regressor (sometimes called the mean model). The dummy regressor is “trained” on the training set simply by memorizing the mean ADD found in the training samples. When presented with new instances, the dummy model always predicts the training mean, regardless of what is provided as input. Therefore the dummy model does not use *X* at all, and is a good minimum benchmark for evaluating the performance of more sophisticated approaches, that presumably extract information from *X* to make predictions.

In the case of the nose data, the dummy regressor outperforms all models shown in Table 3, with a mean absolute error of 77.11 on the testing set, and a NRMSE score of 0.46. While some models achieved a lower mean absolute error in the training phase, the good performance did not extend to the test set.

There were a total of 83 viable samples taken from the ear. The mean ADD for the ear data is 282. Table 4 below summarizes the result of our regression attempts based on data from ear samples alone.

**Table 4:** The ear equivalent of Table 3.

Training error	NRMSE	Regressor	Test error	Dataset
95.89	0.70	SVR	124.62	ORDER_CURATED_EAR
97.24	0.57	SVR	104.33	SPECIES_NONCURATED_EAR
100.01	0.60	SVR	109.60	GENUS_NONCURATED_EAR
100.10	0.60	SVR	120.48	CLASS_CURATED_EAR
100.71	0.56	LR	122.62	CLASS_CURATED_EAR
100.71	0.56	ENR	122.62	CLASS_CURATED_EAR
100.78	0.56	RR	122.52	CLASS_CURATED_EAR
100.94	0.53	RFR	117.59	SPECIES_CURATED_EAR
101.44	0.61	SVR	113.89	FAMILY_NONCURATED_EAR
101.52	0.60	SVR	114.03	ORDER_NONCURATED_EAR

The meaning of Table 4 is similar to that of Table 3. In the case of the ear data, the dummy regressor achieved an error of 165.33 in units of mean absolute error on the test set, and a NRMSE score of 0.68. Unlike the results relating to data harvested from the nose, most models based on the ear data were able to outperform the dummy model. Observe that even though the best model in this category outperformed the dummy model with respect to mean absolute error on the test set, it underperformed with respect to mean squared error, accounting for the slightly higher NRMSE.

While there are data from 83 ear swabs and 69 nose swabs, the number of instances when we achieved quality sequence reads from both ear and nose samples taken from the same cadaver at the same time is 67. The mean ADD of these “joint” samples is 216. Table 5, below, summarizes the results of our regression attempts based on the joint data.

**Table 5:** This table is similar to Table 3, but with joint datasets.

Training error	NRMSE	Regressor	Test error	Dataset
63.74	0.32	KNR	55.02	GENUS_NONCURATED_JOINT
65.49	0.72	SVR	116.22	SPECIES_NONCURATED_JOINT
66.32	0.35	SVR	57.60	GENUS_NONCURATED_JOINT
67.24	0.29	KNR	55.62	ORDER_NONCURATED_JOINT
67.40	0.32	ENR	51.63	SPECIES_NONCURATED_JOINT
67.57	0.32	RR	51.14	SPECIES_NONCURATED_JOINT
68.16	0.42	RFR	53.52	FAMILY_CURATED_JOINT
68.29	0.38	SVR	65.52	ORDER_NONCURATED_JOINT
69.34	0.48	SVR	80.72	FAMILY_NONCURATED_JOINT
69.74	0.35	SVR	62.46	CLASS_NONCURATED_JOINT

The dummy regressor on the joint data had a mean absolute error of 103.31 and a NRMSE score of 0.57. Table 5 shows that in the case of joint data, almost all of the top ten models outperform the dummy regressor by a considerable margin. This improvement comes despite a smaller dataset, indicating a great deal of useful information is gained by simultaneously analyzing the microbiome from both ear and nose swabs.

**Fig 3:**
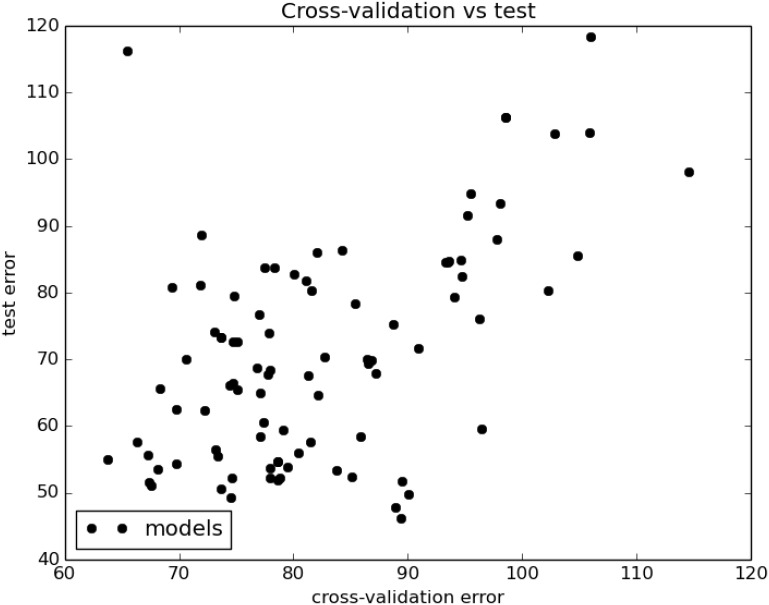
All 91 models considered for the joint data are plotted according to their cross-validation (training) error and test error, in units of mean absolute error. The Pearson *r* = 0.53 with a *p* value of 8.67 × 10^−8^.

Most of the high performing models on the joint dataset use noncurated data from a low taxon. The top model, based on the *k*-neighbors regressor, uses the hyperparameter *k* = 4, and weights the influence of the *k* neighbors by distance. All of the top seven models, excluding the SVR regressor on the species dataset, have a mean absolute error of about 55 on the test set. The surprisingly poor performance of the SVR regressor on the species data (Table 5, row two) may be due to overfitting, abetted by the very high dimensionality of the joint species dataset (more about this below). Indeed, Fig 3 (next section) illustrates that error on the training set is usually a good predictor of test error.

The Pearson coefficient for the correlation between training and test errors is 0.53 with a *p* value of 8.67 × 10^−8^. For our values, the cross-validation error is frequently greater than the test error (*i.e.* testing error). A natural explanation for this is that the 10× cross-validation error results from training on only 90% of the training data, whereas the test error is yielded by a model which has enjoyed the benefit of training on the entire training set. Consequently our cross-validation error is frequently a pessimistic estimate of the test error.

### 4.3 Models Minimizing Test Set Error

All of our analyses described thus far have been under the assumption that our best model is the one with lowest cross-validation error on the training set, for which we offer the associated testing error as an unbiased prediction of the true accuracy of the model. This approach has the virtue that the testing set is not used in any way for model selection, ensuring that the ultimate estimate of accuracy is not optimistic.

**Fig 4:**
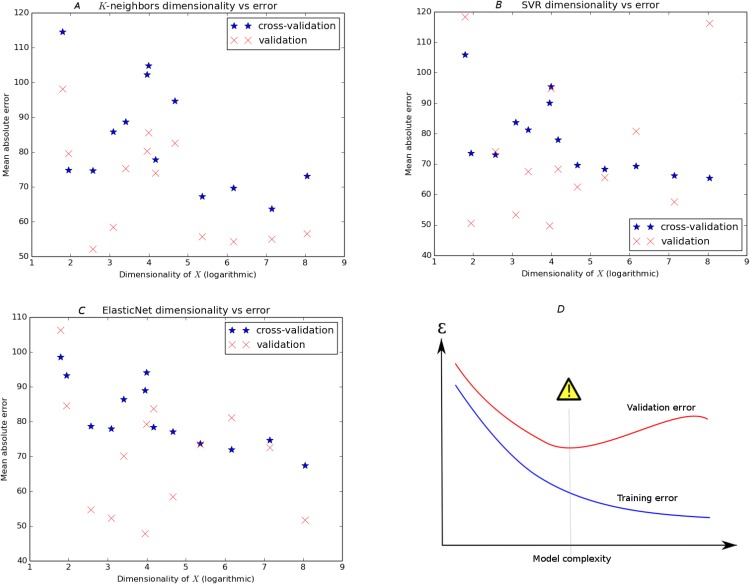
Panel *D* displays the classic diagram for the bias-variance tradeoff, showing how overly complex models minimize training error but may have sub-optimal test error. The other panels show a similar picture for three regressors (SVR, KNeighbors, and ElasticNet) with the dimensionality of the dataset serving as a proxy for model complexity. The horizontal dimension is logarithmic.

However, we next chose to break from conservatively estimating the maximum possible predictive accuracy achievable with our data, and instead we sought to inquire which combination of dataset and regressor is likely to give the best overall generalization based on our data. Here we did not seek an accuracy rate, but instead sought to use our entire dataset for model selection. In this context our test set can be thought of as a final validation set, used for the ultimate validation of a model. For this purpose, we investigated what kinds of models tend to have the lowest errors on our test (*i.e.* validation) set. Fig 4 demonstrates the results of these attempts.

In panel *D* of Fig 4, we show the classic picture of the bias-variance tradeoff. [15] It describes visually how more complex models (models with more parameters, for instance) tend to improve only to a certain level, after which their excessive capacity contributes to overfitting of the training data. Although training error continues to decrease, testing accuracy (the best measure of true performance) increases after the ideal level of complexity is reached. For our models, the dimensionality of the dataset maps well to the complexity of our models, since most parametric regressors include a parameter for each column of *X*. The dimensionality of our data is determined by taxon and whether the data is curated or noncurated (see Table 1).

The other three panels of Fig 4 show, for three regressors (SVR, *k*-Neighbors and elastic net) a similar plot with training error and test error shown on the same axes. In our images, training error tends to be greater than testing error for reasons discussed above, making the pictures somewhat different than the idealized image. However, note that, just as in the diagram, training error decreases as a function of model complexity for our models, while validation error reaches a minimum somewhere in the middle of the domain. Note that the validation error these optimal models achieve is an optimistic representation of accuracy, because we are now using the validation set for the purposes of model selection. Table 6 shows the top ten models when ranked by test error.

**Table 6:** The ten top performing models when ranked by validation error.

Training error	NRMSE	Regressor	Test error	Dataset
89.38	0.26	RR	46.23	PHYLUM_NONCURATED_JOINT
88.93	0.26	ENR	47.82	PHYLUM_NONCURATED_JOINT
74.55	0.30	LR	49.31	SPECIES_NONCURATED_JOINT
90.03	0.27	SVR	49.80	PHYLUM_NONCURATED_JOINT
73.62	0.28	SVR	50.52	PHYLUM_CURATED_JOINT
67.57	0.32	RR	51.14	SPECIES_NONCURATED_JOINT
67.40	0.32	ENR	51.63	SPECIES_NONCURATED_JOINT
89.51	0.29	LR	51.71	PHYLUM_NONCURATED_JOINT
78.66	0.35	RR	51.94	ORDER_CURATED_JOINT
74.66	0.30	KNR	52.17	CLASS_CURATED_JOINT

**Fig 5:**
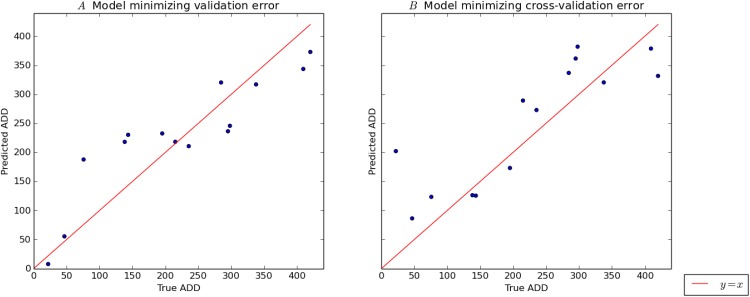
The performance of the best model with respect to validation error on the validation set is described in panel *A*, by plotting true ADD for each element of the test set against the prediction of the model. The identity function is plotted in the same frame for reference. Panel *B* is a similar plot describing the performance of the model which minimized cross-validation error on the training set.

As Table 6 shows, when ranked by performance on the validation set, models based on the phylum taxon perform relatively well. Also, whereas *k*-neighbors was the dominant regressor appearing in Table 5, the models in Table 6 are mainly based on linear regression. The champion regressor, the Ridge algorithm, is simply linear regression with quadratic regularization. Fig 5*A* shows explicitly the performance of this model on the validation set. Fig 5*B* gives a similar description of the best model with respect to training error (see Table 5). It is a happy coincidence that in both Tables 5 and 6, the best regressors are the two simplest algorithms considered.

### 4.4 Univariate Analysis: Finding the Most Informative Taxa

Our analysis reveals that the entire dataset formed a better basis for producing a predictive algorithm for postmortem interval than a curated list of taxa that appear predictive on their own (Tables 1 and 5). This argues that the algorithm is able to glean information from a large number of features that do not appear useful independently. Nevertheless, there is value in knowing which taxa are the most informative for the model that we have constructed. This is because the extreme complexity of a multidimensional approach on the estimation of the postmortem interval comes at the cost of an intuitive understanding of the relationship between certain microbes and decomposition. For this reason, we here include a ranking of various taxa in terms of their relevance to decomposition timing according to several different metrics.

Selecting the best independent variables for predicting a dependent variable is known as feature selection. [22] This task includes the challenge of determining a small but informative subset of features in data like ours in which the number of features is larger than the number of data points. In this study, we have chosen to use a hand-tailored approach for feature selection in the form of our curated data sets and to rely on regularization to eliminate unhelpful features in our non-curated data (Tables 1 and 5). However, here we endeavor to use several feature selection algorithms to rank our features (*i.e.* microbial taxa) in terms of importance. This comes with the important caveat that two or more apparently useless features can, in combination, add accuracy to a regression technique, and that there is no absolute best method for feature ranking. [23]

There are a number of feature selection algorithms, and these differ in several respects such as computational tractability and suitability for specific datasets. Here we consider three methods: F-value, a tree-based approach, and mutual information. The F-value method of feature selection considers the coefficient that results from fitting a single feature with the target using a linear model. It returns a *p*-value that represents the probability that the coefficient is zero, indicating that the feature is useless for linearly modeling the target. The tree-based method ranks features on their tendency to occupy important positions in decision trees built on the same dataset. Finally, the mutual information metric scores each taxon on the amount of information (in the sense of information theory) that it has in common with the dependent variable. The tree and mutual information metrics have the advantage of being able to detect functional relationships that are more complex than simple linear correlation. (Full description of these methods can be found in references [23] and [22] and in the documentation for the Python scikit-learn module, version 0.18, which includes the machine learning primitives we use in our code.) The results of our efforts toward feature selections are summarized in Table 7.

**Table 7:** For each taxon in the leftmost column, this table shows the five most useful organisms for prediction of ADD, as determined by three different ranking methods: F-value, a decision tree based approach, and mutual information. Unless otherwise indicated, terms refer to microbes located in the ear.

Feature Ranking			
	F-value*	Tree	Mutual Information
Phylum	armatimonadetes	verrucomicrobia	actinobacteria
	planctomycetes	armatimonadetes	spirochaetes
	verrucomicrobia	actinobacteria	armatimonadetes
	actinobacteria	planctomycetes	firmicutes†
	cyanobacteria	thermodesulfobacteria†	cyanobacteria†
Class	thermoleophilia	erysipelotrichi	actinobacteria
	erysipelotrichi	clostridia	verrucomicrobiae
	fimbriimonadetes	acidimicrobiia	leptospirae
	planctomycetia	alphaproteobacteria	bacilli
	spartobacteria	solibactereres	thermoleophilia
Order	solirubrobacterales	clostridiales	lactobacillales†
	rhodospirillales	erysipelotrichales	lactobacillales
	caulobacterales	myxococcales	thiotrichales
	myxococcales	xanthomonadales	bacillales
	erysipelotrichales	chthoniobacterales	leptospirales
Family	nocardioidaceae	staphylococcaceae	enterococcaceae
	bradyrhizobiaceae	planococcaceae	staphylococcaceae
	solirubrobacteraceae	enterococcaceae	streptosporangiaceae†
	caulobacteraceae	pseudomonadaceae	planococcaceae
	acetobacteraceae	veillonellaceae	prevotellaceae
Genus	caulobacter	staphylococcus	macrococcus
	acidisphaera	peptoniphilus	vagococcus
	phenylobacterium	vagococcus	symploca†
	dactylosporangium	pseudonocardia	staphylococcus
	nocardioides	oenococcus	pelomonas
Species	virgibacillus salexigens	staph. caprae	actin. rutila
	staph. fleurettii	sporosarcina pasteurii	staph. intermedius
	dactylosporangium maewongense	strep. tigurinus	marinomonas basaltis
	roseomonas terpenica	enterococcus rottae	staph. devriesei
	nocardioides islandensis	staph. epidermidis	propion. granulosum

^†^ located in the nose

* ranked by p-value

As shown in Table 7, each method of feature selection produces a different list of the most powerful features in the data set. Although each of the listed taxa is likely worthy of exploration as a telltale organismal group for the prediction of postmortem interval, our conclusion that analysis of the entire data set, rather than any specific taxon or even a small group of taxa, is the best approach is vindicated by the different results that these methods of feature selection produce.

**Fig 6:**
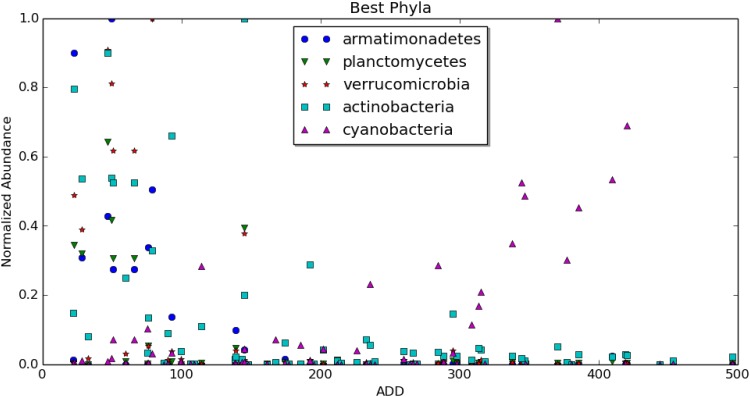
Some select high performing phyla, with ADD plotted against abundance. The vertical axis is normalized for each organism so that the relative abundances are on a similar scale.

Nevertheless, some taxa were identified as powerful indicators by two or even three methods, making these worthy of heightened scrutiny. For example, the phyla actinobacteria and armatimonadetes were identified as top results by all three feature ranking methods, indicating that the collectively considered behavior of organisms within these two phyla had considerable value in our predictive model. Meanwhile, the phyla planctomycetes, verrucomicrobia, and cyanobacteria were identified by two of the three models each, indicating that they have lesser, but still important value in the predictive model (Fig 6).

**Fig 7:**
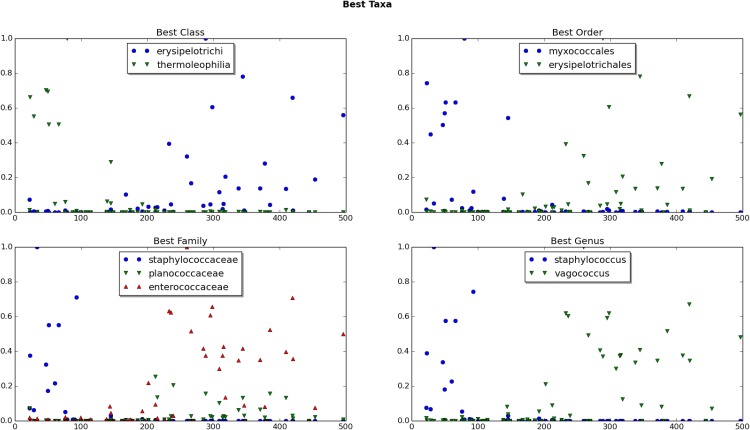
Some select high performing organisms from several taxa, with ADD plotted against abundance. The vertical axis is normalized for each organism so that the relative abundances are on a similar scale.

However, it is important to recall that the total number of phyla in our complete dataset, 52, is relatively small and each encompasses hundreds or even thousands of bacterial species. Therefore, it is not surprising that this would be the taxonomic level at which there is the most agreement among the three methods of feature selection. In fact, the birthday paradox would predict some level of agreement purely by chance. As we moved to lower taxonomic levels, there was far less agreement among the models. This underscores how each taxon likely contributes a small amount of information and the power of the predictive model is found in the consideration of all of the bits of information collectively. The classes thermoleophilia and erysipelotrichi; the orders myxococcales and erysipelotrichales; the families staphylococcaceae, planococcaceae, and enterococcaceae; and the genera staphylococcus and vagococcus were all identified as important features by two of three methods (Fig 7). No species was identified as among the top five indicators by more than one method, underscoring our conclusions that taxonomic levels higher than species are generally the most reliable for construction of a predictive model. This is not to say that species-level information is not important for the construction of the predictive model. Rather, there are no individual species that have much predictive value on their own.

While the results of our feature selection efforts did reveal some bacterial taxa that provide valuable information for our predictive modeling, it is important to bear in mind that the entire data set performs better than a curated collection of even the best performers. This demonstrates the value of a “big data” approach toward building predictive algorithms and the power of regression modeling in extracting valuable information from large amounts of noisy data.

## 5 Conclusions

From this work, we can draw several conclusions. First, the correlation between species diversity (or diversity at other taxa) and ADD is small, but likely real, at least for the ear swabs. In the ear microbiome, diversity tends to be negatively correlated with ADD, whereas it seems to be positively correlated in the nose, where the correlation is less pronounced.

Secondly, our regression attempts based on data from the nose microbes were not successful, and were only marginally successful for data based on the ear microbes alone. However, when the microbiota from both sites were considered jointly, the regression was clearly successful, roughly doubling the accuracy of the dummy regressor, and yielding a model with a mean absolute error of 55 ADD on the validation set. The model that achieved this was simple *k*-nearest-neighbors regression, acting on the genus level data. Furthermore, we showed that regularized linear regression on data at the phylum level may generalize to new instances even better than *k*-neighbors. These are important findings as other postmortem microbial community studies have focused on predictive value at a specific taxonomic level, *e.g.*, genus. [6] Our study highlights the importance of examining taxa at multiple levels simultaneously for predictive value.

55 ADD represents about two days of decomposition during the warm months of summertime in Tennessee and most of the United States. An accuracy of ±2 days in an estimate of the postmortem interval would be a substantial improvement, even in the best of circumstances, over currently available estimation techniques, especially in the interval beyond the reach of forensic entomology.

In comparing our findings to those of similar studies in this area, we find a great deal of corroboration but also significant differences that warrant further study. To date, the most comprehensive examination of decomposition-related microbial succession led to the development of a random-forest regression algorithm for predicting the postmortem interval of a human or mouse cadaver. [24] Although that study reported an impressively thorough characterization and analysis of the microbial ecology of the decomposition niche, the resulting predictive model had an average error rate of 2–3 days limited to the first two weeks of decomposition. Thus, our *k*-nearest neighbor-based model exhibited greater accuracy, and was useful over a longer period of decomposition time, than this previously described method, despite the smaller sample size on which our model was built. It is possible, however, that our small error range may be due, in part, to the homogenous nature of the decomposition environment, as our data all come from one facility. For this reason, a multi-site study is now needed to examine what role, if any, local environment plays in our ability to build a predictive model. It is worth noting, however, that our samples were collected at various points throughout the seasonal year over a period of 14 months.

The use of regression modeling itself has given mixed results in previous studies. For example, Hauther, et al., found that regression analysis was not useful for the construction of a predictive algorithm using necrobiome samples and concluded that the complex ecosystem of decomposition was too noisy for a machine learning approach, at least with only six cadavers to sample from. [12] Instead, they focused on genera known to dominate the human large intestine, from which samples were taken, and found that two of them, Bacteroides and Lactobacillus, displayed a quantitative correlation with the decomposition interval. Neither of these genera were identified as informative by our machine-learning approach, but this is not surprising given that the skin and the intestines are known to harbor vastly different communities of microbes. [1] Use of the necrobiome for the estimation of the postmortem interval has now been explored by a variety of techniques, in a variety of settings, and taken from a variety of sample sites. Our study is unique in that it examined bacteria taken solely from the surface microbiome sampled from the nasal and auditory cavities. We found that neither of these sites, when considered alone, were sufficient for constructing an accurate model using machine learning. However, combining the data led to a dramatic improvement in the predictive modeling. This argues that the skin microbiome holds potential for this potential forensic strategy and that multiple sites should be explored with greater detail in future studies.

As in any machine learning application, our results would improve with the availability of more data. Also, the promised performance estimates on our models depend on new data being drawn from the same statistical distribution as our existing data. For this reason, any new data collected would ideally be in a range of ADD which is of greatest interest to real applications in forensic investigations. Because conjoining the ear and nose data proved successful, it is natural to ask if data collected from an additional site on the cadaver would improve regression accuracy further still. Only a large-scale study at multiple locations and involving several swab sites can resolve these questions.

Nevertheless, we confidently predict that such an effort would produce a predictive algorithm for estimating the postmortem interval for a given decedent with reliable accuracy. Such a tool would be invaluable for forensic investigations throughout the country.
